# Jejunal perforation during peritoneal dialysis catheter placement: A case report

**DOI:** 10.1016/j.amsu.2020.07.012

**Published:** 2020-07-18

**Authors:** Sukit Raksasuk, Woraboot Taweerautchana, Thatsaphan Srithongkul

**Affiliations:** a Division of Nephrology, Department of Medicine, Faculty of Medicine Siriraj Hospital, Mahidol University, Bangkok, Thailand; b Department of Surgery, Faculty of Medicine Siriraj Hospital, Mahidol University, Bangkok, Thailand

**Keywords:** Case report, Peritoneal dialysis catheter, Bowel perforation, Peritoneal dialysis, Catheter placement

## Abstract

**Introduction:**

Bowel perforation is a rare but serious complication after peritoneal dialysis (PD) catheter insertion, which significantly increases mortality. Currently, there is no recommendation for preferring catheter insertion technique, since neither open surgical or percutaneous technique demonstrate superior outcome.

**Presentation of case:**

This is a 78-year-old man who developed jejunal perforation during PD catheter placement, presenting with initial clear and satisfying PD fluid drainage. Bowel perforation was recognized after long dwell of PD fluid returned in yellowish color. Operative finding revealed a through and through jejunal wall perforation.

**Conclusion:**

Satisfying dialysate flow and tip catheter location could not exclude accidental bowel perforation after PD catheter placement. Carefully patient monitoring is crucial in detecting postoperative complication.

## Highlights

• Satisfying dialysate flow and tip catheter location could not exclude bowel perforation after PD catheter placement.
• Long dwell of peritoneal dialysate increase sensitivity for detecting bowel injury after PD catheter insertion.
• Early detection and treatment of bowel injury after PD catheter placement is crucial to minimize the patient morbidity.

## 1 Introduction

Currently, the use of peritoneal dialysis (PD) has been increased among HIV patients who have met the criteria for renal replacement therapy. There are several techniques for PD catheter implantation, including open surgery, percutaneous Seldinger technique, and laparoscopic surgery [1]. One of the intra-abdominal organ injuries, bowel perforation, is a serious complication and needs early detection and prompt management. There is no consensus for preferring PD catheter placement techniques regarding each method could not provide superior outcome [2]. We reported the case of end stage renal disease (ESRD) patient, who developed jejunal perforation after PD catheter insertion by percutaneous Seldinger's technique. Our case has been reviewed and reported in line with the SCARE 2018 guidelines [3].

## 2 Case presentation

A 78-year-old man with chronic kidney disease (CKD) stage 5 presented with acute dyspnea and productive cough for 1 week. He had HIV infection for 10 years and currently on highly active antiretroviral therapy (HAART). He was diagnosed *Klebsiella* pneumonia with respiratory failure. After admission, he developed massive upper GI bleeding and profound shock, led to cardiac arrest. After resuscitation, he gained a return of spontaneous circulation but still had oliguria. Continuous renal replacement therapy (CRRT) was initiated via right femoral non-cuff catheter for metabolic and volume control. After that, his hemodynamic was improved, and CRRT was withdrawn. Since he had had CKD stage 5, PD catheter placement was made for long term renal replacement therapy. He did not have any previous abdominal surgery or other contraindication for PD.

Laboratory tests on the day of operation were consisted with ESRD as following: serum urea nitrogen 81 mg/dL; serum creatinine 6.2 mg/dL, corresponding to an estimated glomerular filtration rate of 5 ml/min/1.73m^2^ (as calculated by CKD-EPI [Chronic Kidney Disease Epidemiology Collaboration] equation); serum sodium 143 mEq/L; serum potassium 3.8 mEq/L; serum chloride 103 mEq/L; serum bicarbonate 17 mEq/L; hemoglobin 10.5 g/dL, white blood cell count, 10980/μL; platelet count, 168,000/μL; serum albumin 2.3 g/dL.

The patient was given lactulose 30 mL for bowel preparation for pre-operative preparation. On the operation day, the patient was sedated with fentanyl 50 μg intravenously.

**Fig. 1:**
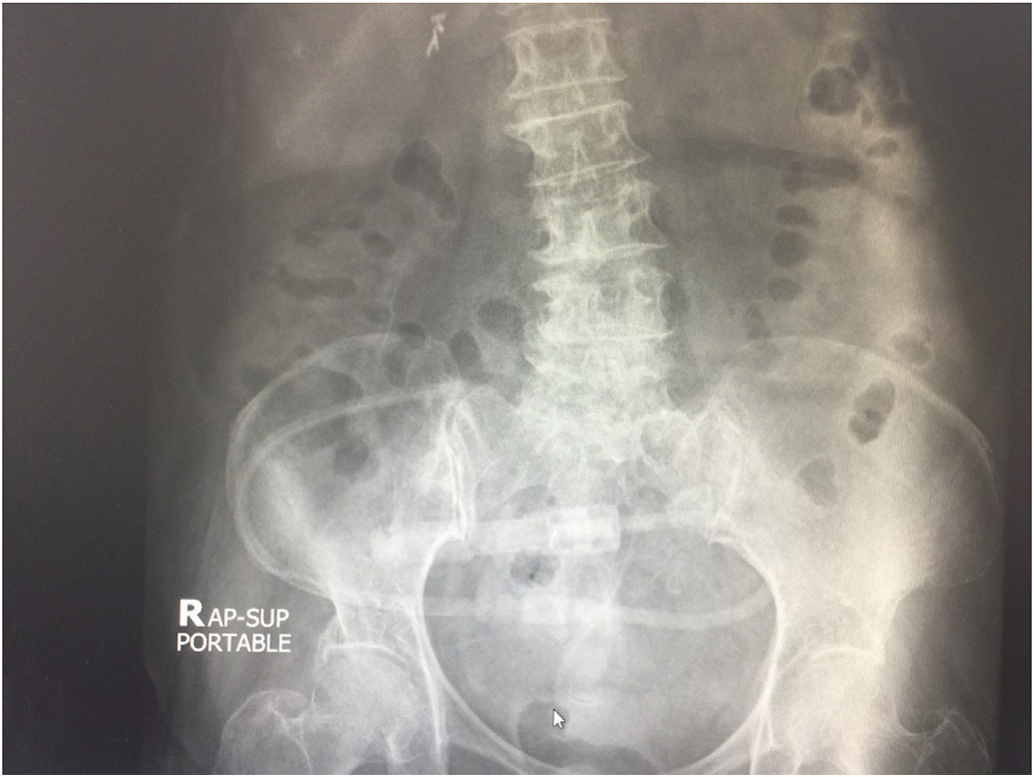
An abdominal X-ray showed an optimal position of the PD catheter.

**Fig. 2:**
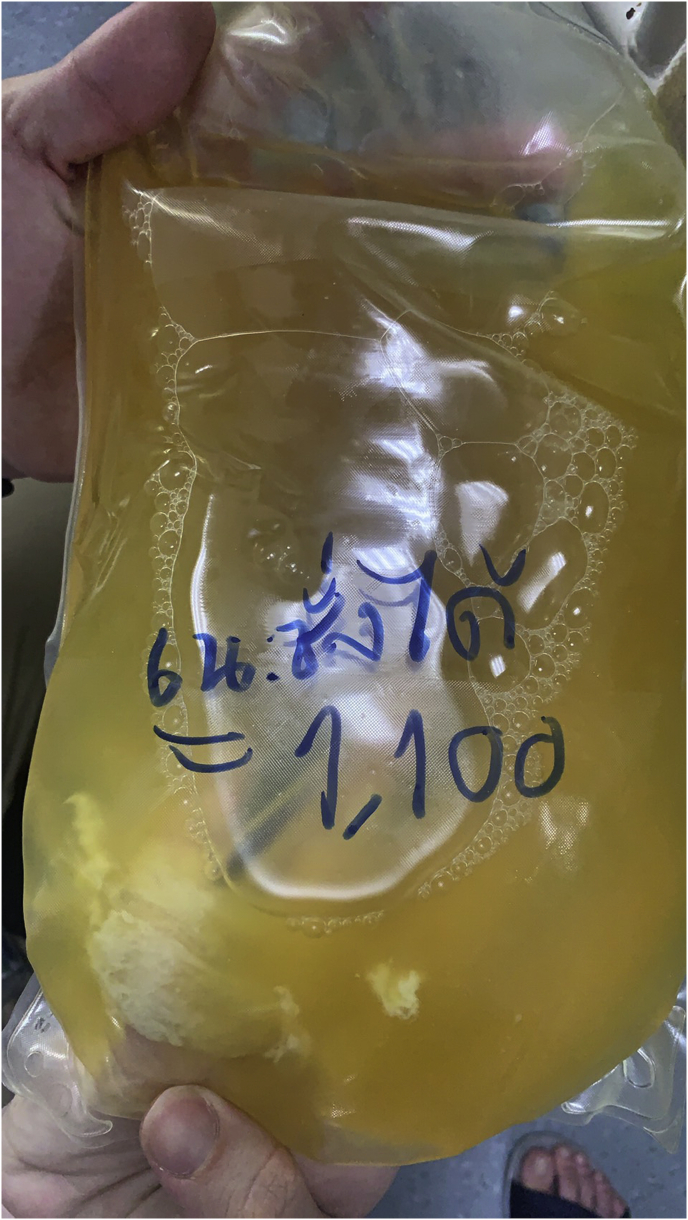
Yellowish color PD effluent with small fibrin after the first 4 hours dwell. (For interpretation of the references to color in this figure legend, the reader is referred to the Web version of this article.)

**Fig. 3:**
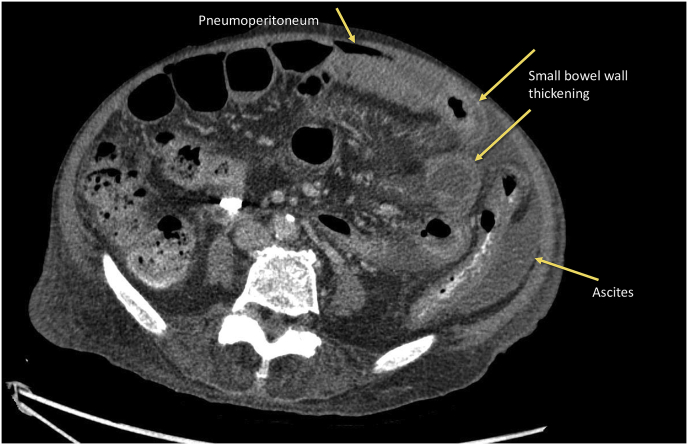
Abdominal CT scan demonstrated small pneumoperitoneum with air bubbles trapped in pelvic fluid.

**Fig. 4:**
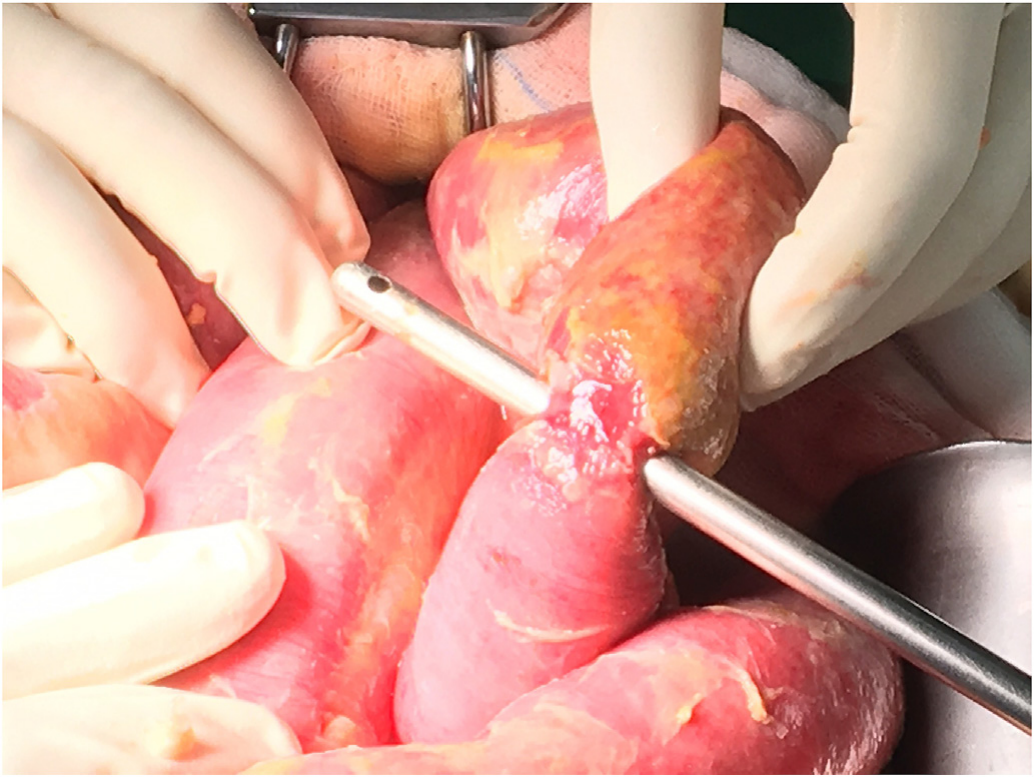
A through-and-through jejunal wall perforation.

He underwent PD catheter insertion by interventional nephrologist under local anesthesia at the bedside, despite unable transferring to the operating theater. The midline sub-umbilical incision and subcutaneous fat dissection were performed to reach the rectus sheath. The introducing sharp 16 gauge needle was applied one attempted, and normal saline 500 ml was instilled. A 150 cm. guide wire was introduced using the Seldinger technique. The PD catheter was carefully advanced into the peritoneal cavity as deep as usual with a deep cuff within the rectus sheath. One liter of normal saline was instilled through the peritoneal cavity then minimal inactive bloody color fluid was observed. Both inflow and outflow rates were satisfied. An abdominal X-ray showed an optimal position of the PD catheter (Fig. 1). In the immediate postoperative period, his clinical was stable. A rapid 1-liter exchange was continued every 1 hour, and the effluent showed adequate drainage during the first six hours. However, the effluent became lightly yellowish color and contained small fibrin after the first 4 hours dwell, as depicted in Fig. 2. The patients developed a high-grade fever at 12 hours after the operation. He was normotensive and denied any abdominal pain. Complete blood cell count showed a hemoglobin level of 10.6 g/dL, white blood cell count of 12,000/μL, and platelet count of 207,000/μL. PD fluid was sent for cell count, which showed white blood cell 1750 cell/μL with 92% of neutrophil. The PD fluid gram stain revealed gram-negative rods, so the intestinal perforation was suspected. The emergency abdominal computerized tomography scan (CT scan) was performed in this case for rule out any iatrogenic hollow viscus organ injury because of equivocal peritoneal sign on abdominal exam. Abdominal CT scan demonstrated small pneumo-peritoneum with air bubbles trapped in pelvic fluid and thickening of the segmental small-bowel wall in the left-sided abdomen (Fig. 3). The patient was transferred to the operating theater for an emergency exploratory laparotomy. There was no inadvertent movement of the catheter and it was secured properly. Operative finding revealed 100 ml of bile content and pus in the abdominal cavity. Moreover, a through-and-through jejunal wall perforation was identified (about 50 cm from the ligament of Treitz), as depicted in Fig. 4. The perforated sites were closed by 3-0 Vicryl interrupted sutures. After that, the abdominal toilet was done properly, and the abdominal wall was closed as usual fashion. Finally, the patient was transferred to the ICU in stable condition. Postoperatively, intravenous meropenem was given for the empirical treatment and was discontinued on postoperative day 7 in regards to the negative result of blood culture. After surgery, the hemodynamic was stable, and normal bowel movement was observed in 48 hours. Intermittent hemodialysis was re-started without any complication during dialysis treatment. Hemodialysis was considered for long term renal replacement therapy for this patient.

## 3 Discussion

Bowel perforation was a rare but serious complication of PD catheter placement [4]. The previous study demonstrated that bowel perforation increased mortality in PD patients [5]. We performed total 278 PD catheter implantations from 2010 to 2020. Twenty-three cases underwent laparoscopic method, 13 cases underwent percutaneous Seldinger method, and 242 cases underwent conventional open methods. Among 242 patients implanted using conventional open methods, 2 cases had bowel perforation. However, we did not experience any complications, among other techniques. As previous systematic review demonstrated that no different outcomes associated surgical and infectious complications among peritoneal catheter placement techniques [6]. We chose the open approach with close puncture technique under local anesthesia in this patient regarding to the open approach or close puncture technique under local anesthesia in very frail patients is safer than laparoscopic approach because it results in less hemodynamic effect compare to laparoscopic approach (pneumo-peritoneum compresses on IVC).

Regarding the serious complications of PD catheter placement, bowel perforation had been recognized varying upon insertion techniques. The incidence of internal organ injury was 1.0–1.4% by conventional open technique but lack of data in the actual incidence in Seldinger's technique [1,7]. Previous reports showed 1% of the patient developed bowel perforation during initiated guide wire or advancing stylet entry into the abdominal cavity [8,9]. The characteristics and sites of bowel injuries were determined on the location of catheter implantation [12]. The small bowel perforation may occur when placing the catheter in the midline location. As in our patient, the catheter was implanted in the midline by Seldinger's technique. Initially, we decided to dissect and identify the posterior rectus sheath before applying an introducing needle in order to avoid any possibility of internal organ injury. Unfortunately, unrecognized bowel perforation occurred during the procedure. Because of an accidental punctured through-and-through jejunal wall, consequence the guide wire and catheter were into the lumen.

Several studies showed the risk factors associated with this complication, including previous surgery and intra-abdominal adhesion [10,11]. We believe that the possible cause of this complication was the patient developed marked bowel dilatation due to bowel ileus. Therefore, the possibility of iatrogenic bowel puncture was higher than usual situation. Retrospectively, abdominal wall lifting technique using two towel clips before we punctured the needle or changing to true open approach (abdominal wall was carefully opened layer by layer until we visually entered into the abdominal cavity) might reduce the possibility of this complication.

Satisfying drainage and no feculent effluent was observed during a previous exchange as a result of the catheter tip was placed in the pelvic cavity. Several previous studies defined the criteria for diagnosis bowel injury during PD catheter implantation, including feculent brownish color drainage, or watery diarrhea [12]. We did not notice the bowel perforation in our patient until we obtained the brownish color of the long dwell dialysate. Therefore, a learning point of this case is that satisfying dialysate flow and tip catheter location could not exclude accidental bowel perforation after PD catheter placement.

## 4 Conclusion

We suggest that early detection and treatment of bowel injury after PD catheter placement is crucial in order to minimize the morbidity and mortality rate of the patients.

## Ethics approval

Not applicable regarding one case report.

## Funding

None.

## Author contribution

Dr. Raksasuk conceptualized, drafted, reviewed and revised the manuscript. Dr. Srithongkul contributed to the concept of the report, drafted,critically reviewed and revised the manuscript. Dr.Taweerautchana reviewed the manuscript. All authors approved the final manuscript as submitted and agree to be accountable for all aspects of the work.

## Registration of research studies

1. Name of the registry:
2. Unique identifying number or registration ID:
3. Hyperlink to your specific registration (must be publicly accessible and will be checked):

## Guarantor

Thatsaphan Srithongkul.

## Consent of patient

Written informed consent was obtained from the patient for publication of this case report and accompanying images. A copy of the written consent is available for review by the Editor-in-Chief of this journal on request.

## Availability of data and materials

Further clinical data of this case are available from the corresponding author upon reasonable request.

## Provenance and peer review

Not commissioned, externally peer reviewed.

## Declaration of competing interest

All authors have no relevant financial interests or conflicts of interest to report.
